# Supplementary material for: A comparative study of bacterial diversity based on effects of three different shade shed types in the rhizosphere of Panax quiquefolium L
Source: PeerJ. 2022 Feb 9;10:e12807. doi: 10.7717/peerj.12807 (PMC8840058; doi:10.7717/peerj.12807)
Supplement: Supplemental Information 1 [file peerj-10-12807-s001.docx]

| Shade type | pH | Organic matter (g/kg) | Hydrolyzable nitrogen (mg/kg) | Available phosphorus (mg/kg) |
| --- | --- | --- | --- | --- |
| GP | 6.35±0.04a | 16.1±0.30a | 177.0±1.37a | 70.7±1.80a |
| PP | 4.98±0.03a | 20.8±0.36b | 165.4±2.09b | 116.8±1.25b |
| CTP | 5.07±0.04b | 18.7±0.17c | 190.8±1.80c | 76.8±1.28c |

* Different letters indicate significant differences between treatments at the 0.05 level.
