# Supplementary material for: A comparative study of bacterial diversity based on effects of three different shade shed types in the rhizosphere of Panax quiquefolium L
Source: PeerJ. 2022 Feb 9;10:e12807. doi: 10.7717/peerj.12807 (PMC8840058; doi:10.7717/peerj.12807)
Supplement: Supplemental Information 2 [file peerj-10-12807-s002.docx]

| Shade type | Fresh weight (kg) | number of plants per acre | [increase](C:/Users/GX/AppData/Local/Youdao/Dict/Application/8.9.4.0/resultui/html/index.html#/javascript:;) [production](C:/Users/GX/AppData/Local/Youdao/Dict/Application/8.9.4.0/resultui/html/index.html#/javascript:;)  (%) | Diseased plant number per acre | Diseased plant weight  (kg/acre) | Diseased incidence  (%) | Disease severity index (%) |
| --- | --- | --- | --- | --- | --- | --- | --- |
| GP | 495.6±13.55a | 25419±695.50a | 12.7 | 910±21.50a | 19.5±0.45a | 3.58 | 10.5 |
| PP | 466±13.40a | 20535±589.90b | 6 | 1066±82.07b | 20.2±0.93b | 5.19 | 9.8 |
| CTP | 439.3±9.70b | 23865±527.04c | -- | 3019±196.52c | 44.0±2.87c | 12.65 | 18.6 |

* Different letters indicate significant differences between treatments at the 0.05 level.
