# Supplementary figures and images for: A comparative study of bacterial diversity based on effects of three different shade shed types in the rhizosphere of Panax quiquefolium L
Source: PeerJ. 2022 Feb 9;10:e12807. doi: 10.7717/peerj.12807 (PMC8840058; doi:10.7717/peerj.12807)

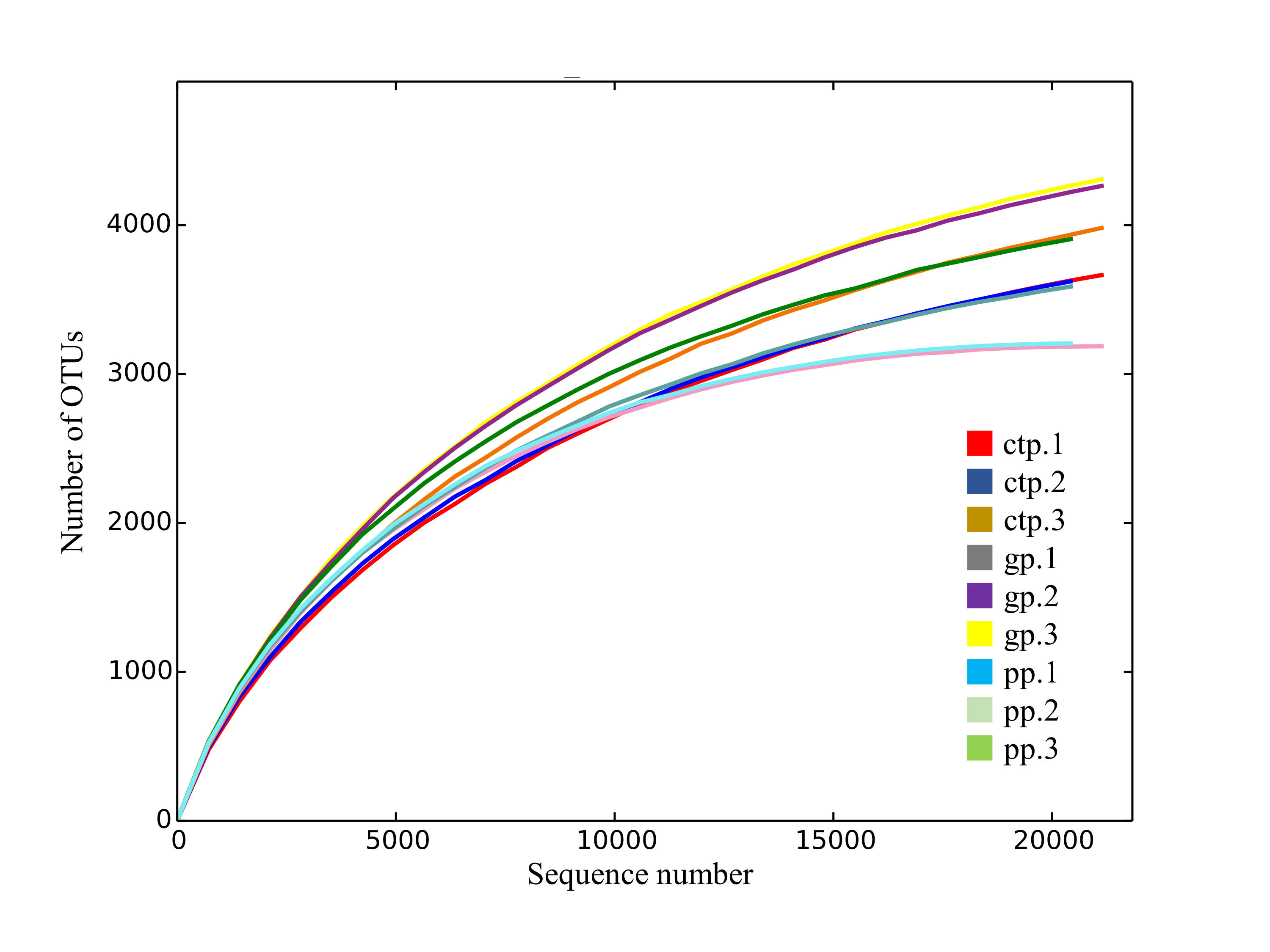

Supplement: Supplemental Information 3 [file peerj-10-12807-s003.png]

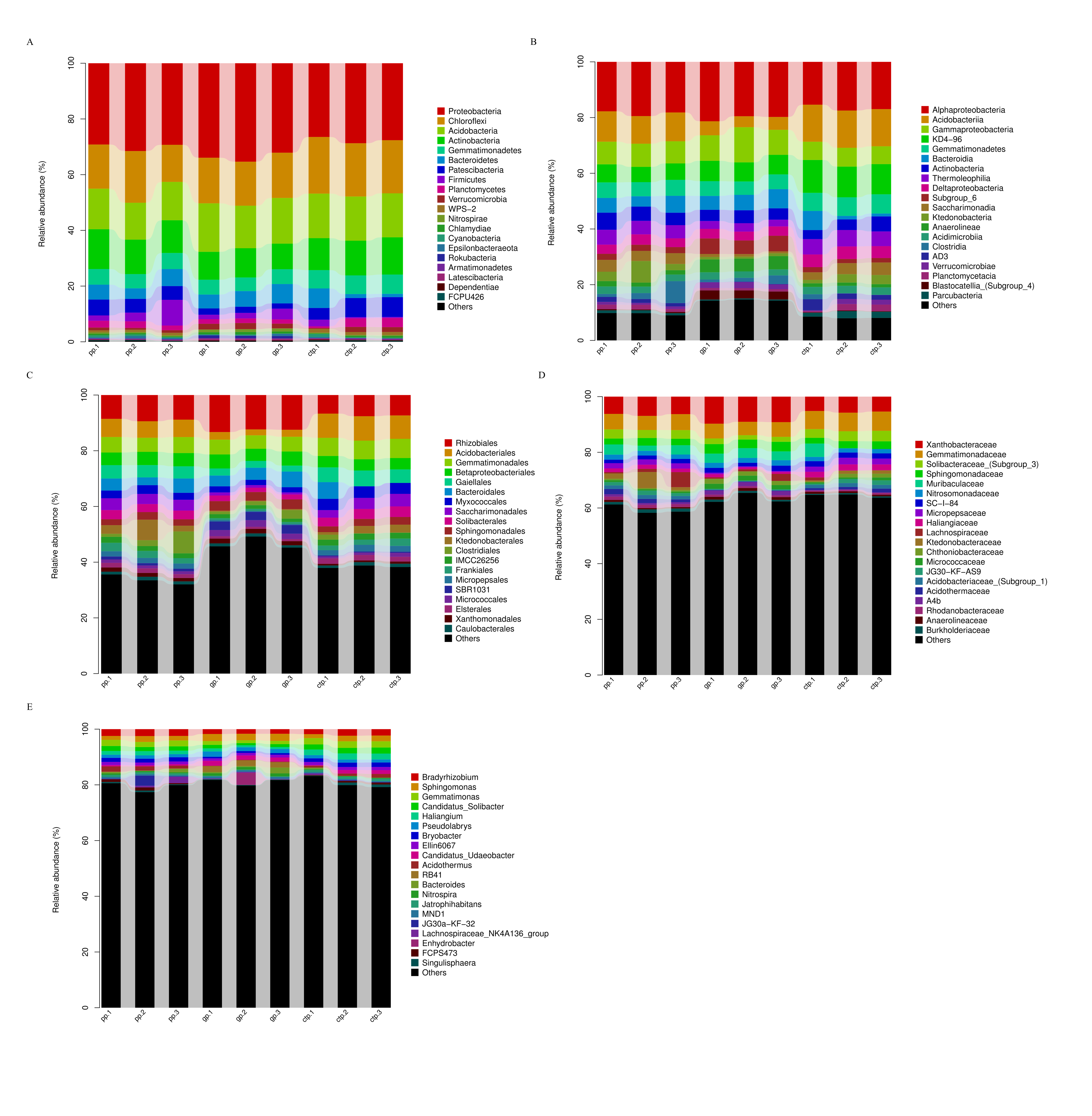

Supplement: Supplemental Information 4 — (A) Phylum. (B) Class. (C) Order. (D) Family. (E) Genus. [file peerj-10-12807-s004.png]

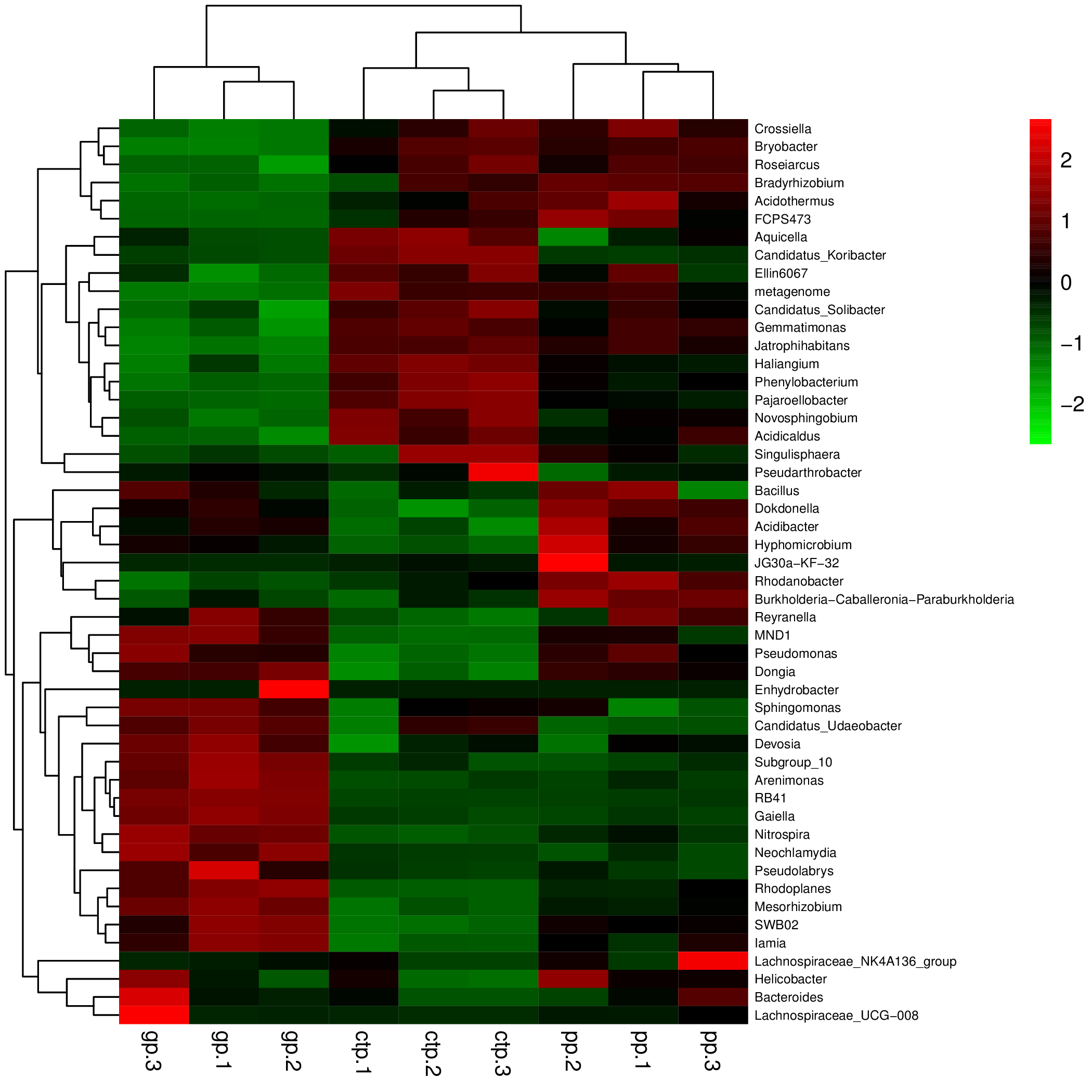

Supplement: Supplemental Information 5 [file peerj-10-12807-s005.png]

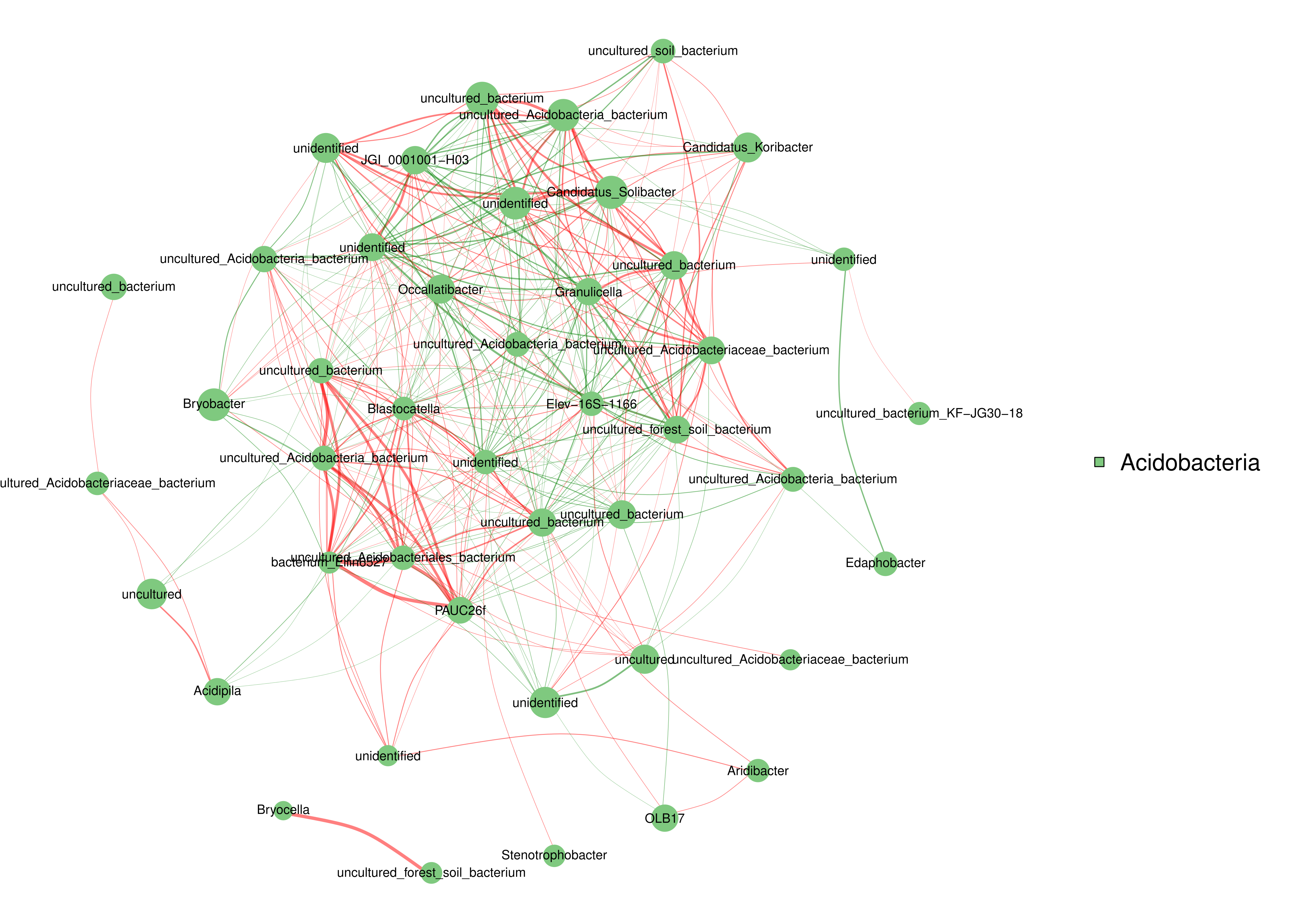

Supplement: Supplemental Information 6 [file peerj-10-12807-s006.png]
