# Supplementary material for: A comparative study of bacterial diversity based on effects of three different shade shed types in the rhizosphere of Panax quiquefolium L
Source: PeerJ. 2022 Feb 9;10:e12807. doi: 10.7717/peerj.12807 (PMC8840058; doi:10.7717/peerj.12807)
Supplement: Supplemental Information 7 [file peerj-10-12807-s007.docx]

| Name | Explains % | pseudo-F | P |
| --- | --- | --- | --- |
| pH | 56.8 | 9.2 | 0.016 |
| H-N | 16.0 | 3.5 | 0.002 |
| A-P | 3.9 | 0.8 | 0.57 |
| TOC | 2.8 | 0.5 | 0.702 |
